# Hepatic arterial infusion chemotherapy followed by sorafenib in patients with advanced hepatocellular carcinoma (HICS 55): an open label, non-comparative, phase II trial

**DOI:** 10.1186/s12885-018-4519-y

**Published:** 2018-06-04

**Authors:** Masahiro Hatooka, Tomokazu Kawaoka, Hiroshi Aikata, Yuki Inagaki, Kei Morio, Takashi Nakahara, Eisuke Murakami, Masataka Tsuge, Akira Hiramatsu, Michio Imamura, Yoshiiku Kawakami, Kazuo Awai, Keiichi Masaki, Koji Waki, Hirotaka Kohno, Hiroshi Kohno, Takashi Moriya, Yuko Nagaoki, Toru Tamura, Hajime Amano, Yoshio Katamura, Kazuaki Chayama

**Affiliations:** 1 Department of Gastroenterology and Metabolism, Institute of Biomedical & Health Science, Hiroshima University, Hiroshima, 734-8551 Japan; 2 Department of Diagnostic Radiology, Graduate School of Biomedical Sciences, Hiroshima, 734-8551 Japan; 3 Hiroshima City Asa Hospital, Hiroshima, Japan; 4 Kure Medical Center, Hiroshima, Japan; 5 Chugoku Rousai Hospital, Hiroshima, Japan; 6 Mazda Hospital, Hiroshima, Japan; 7 Onomichi General Hospital, Hiroshima, Japan; 8 Liver Research Project Center, Hiroshima University, Hiroshima, Japan; 9 Laboratory for Digestive Diseases, RIKEN Center for Integrative Medical Sciences, Hiroshima, Japan

**Keywords:** HCC, HAIC, Sorafenib, Tumor marker, RECIST

## Abstract

**Background:**

In patients with advanced hepatocellular carcinoma (HCC), evidence is unclear as to whether hepatic arterial infusion chemotherapy (HAIC) or sorafenib is superior. We performed a prospective, open-label, non-comparative phase II study to assess survival with HAIC or HAIC converted to sorafenib.

**Methods:**

Fifty-five patients were prospectively enrolled. Patients received HAIC as a second course if they had complete response, partial response, or stable disease (SD) with an alpha fetoprotein (AFP) ratio < 1 or a des-γ-carboxy prothrombin (DCP) ratio < 1. Patients were switched to sorafenib if they had SD with an AFP ratio > 1 and a DCP ratio > 1 or disease progression. The primary endpoint was the 1-year survival rate. Secondary endpoints were the 2-year survival rate, HAIC response, survival rate among HAIC responders, progression-free survival, and adverse events.

**Results:**

Of the 55 patients in the intent-to-treat population, the 1-year and 2-year survival rates were 64.0 and 48.3%, respectively. After the first course of HAIC, one (1.8%) patient showed complete response, 13 (23.6%) showed partial response, 30 (54.5%) had SD, and 10 (18.1%) patients had progressive disease. Twenty-three patients (41.8%) had SD with AFP ratios < 1 or DCP ratios < 1, and 7 (12.7%) had SD with AFP ratios > 1 and DCP ratios > 1. Thirty-seven patients (68.5%) were responders and 17 (30.9%) were non-responders to HAIC. In responders, the 1-year and 2-year survival rates were 78 and 62%, respectively.

**Conclusion:**

Given the results of this study, this protocol deserves consideration for patients with advanced HCC. This trial was registered prospectively from December 12. 2012 to September 1. 2016.

## Background

Hepatocellular carcinoma (HCC) is the sixth most common cancer and the second leading cause of cancer-related mortality in the world. [1, 2]. Advances in technology have contributed to development of new diagnostic techniques such as ultrasonography, computed tomography, magnetic resonance imaging, and angiography. Similarly, new treatment modalities have been developed, including surgical resection, radiofrequency ablation [3], percutaneous ethanol injection, transcatheter arterial chemoembolization (TACE), and hepatic arterial infusion chemotherapy (HAIC), resulting in improved prognosis in HCC patients [4–12]. However, the survival rates are still poor for patients with advanced HCC with associated complications such as portal vein tumor thrombosis, and refractoriness to TACE.

Two phase III clinical trials of sorafenib for advanced HCC showed significant efficacy in terms of overall survival (OS) time compared with placebo [13, 14]. Based on these studies, sorafenib has become the standard of therapy for advanced HCC. Sorafenib is associated with extension of OS time by 2.3–2.8 months and the improvement of response rate by 2.0–3.3%. However, the survival advantage of sorafenib has been described as insufficient.

HAIC is widely used throughout Asia, especially in Japan. Several studies have shown the survival benefits of HAIC for advanced HCC free of extrahepatic metastasis (extrahepatic spread, or EHS), with response rates ranging from 20.8 to 52%, and have shown that the median survival time (MST) in responders ranges from 17.6–40.7 months [11, 12, 15–18]. In most retrospective studies, the survival time was much better among responders than non-responders. Nevertheless, HAIC is not regarded as the standard of care for advanced HCC patients as no prospective randomized phase III trials have shown survival benefits in patients with advanced HCC.

Among responders, a better prognosis was expected with HAIC compared with sorafenib, while HAIC non-responders had a poor prognosis at 6 months in previous studies. Therefore, it is necessary to identify HAIC non-responders as early as possible.

In a previous study, we reported that patients showing either complete or partial response (CR and PR respectively) by the first course of HAIC had good prognoses, whereas patients with progressive disease [19] by the first course of HAIC had poor prognoses. However, we observed that the majority of patients had stable disease (SD) after the first course of HAIC. Furthermore, we reported that among patients determined to have SD based on the imaging response to the first course of HAIC, those with alpha fetoprotein (AFP) and des-γ-carboxy prothrombin (DCP) ratios > 1 had significantly poorer survival times [20].That is, patients in whom AFP or DCP levels decreased had better prognoses than those in whom AFP or DCP levels increased. Therefore, we considered patients to be HAIC responders in the first course of HAIC when they showed CR, PR, or SD with decreased levels of AFP or DCP. We defined HAIC non-responders as either patients with PD or patients with SD who had increased levels of AFP and DCP after the first course of HAIC.

Few prospective studies of HAIC have been performed. No study protocols have been examined in which HAIC was continued only in responders while non-responders were switched to sorafenib where the outcome of the first course of HAIC was determined by early assessment of tumor markers and imaging responses. Therefore, we created a protocol in which HAIC was continued unless the outcome of therapy was non-response, and non-responders were then switched from HAIC to sorafenib.

## Methods

### Study design

The phase II HICS study (Hepatic Arterial Infusion Chemotherapy followed by Sorafenib) was a single-arm, prospective, open-label trial. In this study, the primary endpoint was the survival rate at 1 year. The secondary endpoints were the survival rate at 2 years, overall survival (OS), response to HAIC, survival rate according to HAIC response, progression-free survival (PFS), and adverse events (AEs). The primary endpoint, survival rate at 1 year, was defined as the probability of patients being alive 1 year after their first course of HAIC. OS was defined as the time from the start of the study treatment to the death due to any reason. PFS was defined as the time from the start of study treatment to the first documentation of objective tumor progression or to death due to any cause.

One month after the first course of HAIC, therapeutic efficacy was assessed by imaging studies and AFP/DCP. Results of imaging studies were assessed according to the Response Evaluation Criteria In Solid Tumors.

Safety assessments of the drugs included recording of AEs, changes in laboratory test results, physical examination, and vital signs. Adverse events associated with the drugs were those listed in the Common Terminology Criteria for Adverse Events (CTCAE) 4.0.

The study was registered with the University Hospital Medical Information Network Clinical Trials Registry as HICS 55, with the identifier number UMIN 000009094. The study was approved by the ethics committee and conducted in accordance with the Declaration of Helsinki. Informed consent was obtained from each patient.

### Patients

Key inclusion criteria were as follows: minimum age of 20 years; life expectancy of at least 12 weeks at the pre-treatment evaluation; advanced HCC based on histological evidence via biopsy specimen or dynamic computed tomography or magnetic resonance imaging; not eligible for resection or local ablation therapy or TACE; at least 4 weeks since the last therapy for HCC; no prior sorafenib and HAIC treatment; no intrahepatic tumor that could affect patient prognosis; Eastern Cooperative Oncology Group performance status of 0 or 1; Child-Pugh score of 5, 6, or 7; and adequate bone marrow, liver, and renal function, as assessed by the following laboratory requirements: granulocyte count ≥3000/mm^3^, platelet count ≥50,000 /mm^3^, hemoglobin ≥8.5 g/dL, total serum bilirubin ≤3 mg/dL, serum albumin ≥2.8, serum creatinine ≤1.5 mg/dL, prothrombin consumption test ≥50%, and amylase ≤ twice the upper limit of normal. Key exclusion criteria were as follows: other malignant disease, pregnancy or suspected pregnancy, severe infectious disease, history of severe allergy, severe renal function disease, severe allergy to 5-fluorouracil or cisplatin, severe bone marrow suppression, esophageal and/or gastric varices with a high risk of bleeding and clinically significant gastrointestinal bleeding, or serious hypertension. Patients who were unstable or whose safety or compliance in the study could be jeopardized based on the investigator’s judgment were also excluded.

### Treatments

**Fig. 1:**
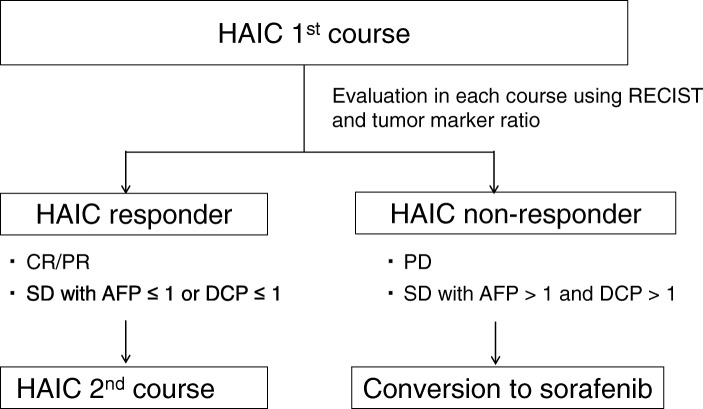
Study schema. Abbreviations: *AFP* alpha fetoprotein, *CR* complete response, *DCP* des-gamma-carboxy prothrombin, FP, *HAIC* hepatic arterial infusion chemotherapy, *PD* progressive disease, *PR* partial response, *SD* stable disease

Figure 1 illustrates the study schema. HAIC was administered as the first therapy. Within one month after HAIC administration, efficacy was assessed by imaging studies and AFP/DCP. Patients who showed CR or PR or SD with AFP ratio < 1 or DCP ratio < 1 were defined as responders. Patients who showed SD with AFP ratio > 1 and DCP ratio > 1 or PD [19] were defined as non-responders. Responders continued HAIC while non-responders were switched from HAIC to sorafenib. The therapeutic efficacy of sorafenib was assessed by imaging studies and AFP/DCP one month after starting therapy. TACE was provided to partial and non-responders during this study.

### Hepatic arterial infusion chemotherapy

Cisplatin was administered at a dose of 20 mg/m^2^/day on days 1 and 8, and fluorouracil was administered at a dose of 330 mg/m^2^/day on days 1–5 and 8–12 of every 28-day cycle, followed by 2 weeks off. HAIC was interrupted in patients who experienced hematologic and non-hematologic toxicities attributed to HAIC.

### Sorafenib

Sorafenib 400 mg bid was used for the treatment of patients who switched from HAIC. Sorafenib doses were adjusted, by interruption or reduction, in patients who experienced clinically significant hematologic or non-hematologic toxicities attributed to sorafenib. Sorafenib doses were reduced stepwise from 400 mg twice daily to 400 mg once daily to 400 mg every other day to 200 mg every other day as warranted. Stepwise increases were allowed after resolution of the AE. TACE, radiation therapy, and hepatectomy were allowed as additional therapies.

### Statistical analysis

We assumed a threshold survival rate at 1 year of 45% with an expected survival rate at 1 year of 60% (0.1 α-error and 0.1 β-error). From these, we predicted that 46 patients would qualify and established a patient enrollment target of 55 assuming that 20% would be disqualified.

Statistical analysis was performed using SPSS (IBM, Armonk, NY, USA). Continuous variables are expressed as medians and ranges, while categorical variables are expressed as counts or frequencies. Kaplan–Meier survival curves with log-rank tests were used for the analysis of OS. The statistical analysis was performed in September 2017. Differences between groups were examined for statistical significance using the Mann-Whitney U test, logistic regression test, or chi-square test as appropriate. The cumulative survival rate was calculated from the date of initiation of HAIC and assessed by the Kaplan-Meier life-table method. Differences between groups were evaluated by the log-rank test. For baseline characteristics such as performance status, age, stage of disease, and history of therapy, we calculated frequencies, averages, and medians to assess their distribution.

Variables that achieved statistical significance (*P* < 0.05) or marginal significance (*P* < 0.10) in the univariate analysis were entered into multiple logistic regression analysis to identify significant independent predictive responders.

Multivariate Cox proportional hazards regression was performed to assess the independent prognostic factors. For both univariate and multivariate analyses, all independent factors that demonstrated statistical significance as a predictor were analyzed using stepwise selection in the model. Hazard ratios and corresponding 95% confidence intervals are reported.

## Results

### Baseline characteristics

Between December 2012 and October 2016, 55 patients with unresectable HCC were enrolled in this study at participating hospitals in the Hiroshima Liver study group. The median period of observation was 12.2 months with a range of 2.1 to 54.6 months. The data was last updated on September 2017.

Patient characteristics are listed in Table 1. The majority of study subjects were male, with a median age of 66 years. Among 29 patients who had Vp 3 and 4, 19 patients received three-dimensional conformal radiotherapy. Patients received HAIC therapy a median of two times (range: 0 to 11 times).

**Table 1:** Background characteristics of patients who received hepatic arterial infusion chemotherapy

Characteristics	Median (range) or patient numbers
Age (years)	66 (32–88)
Gender (M/F)	49/6
ECOG performance status (0/1)	50/5
Etiology (HBV/HCV/others)	14/24/17
Platelet count (/mm^3^)	15.6 (6.4–41.4)
Total bilirubin (mg/dL)	0.9 (0.3–1.8)
Albumin (g/dL)	3.7 (2.7–5.0)
Prothrombin consumption test (%)	78 (57.4–118)
Child-Pugh score (5/6/7)	22/23/10
Number of liver tumors	5 (1–40)
Size of liver tumors (mm)	85 (18–170)
Macroscopic vascular invasion (without/with)	17/38
Vp (0–2/3–4)^a^	26/29
Vv (0–1/2–3)	46/9
Relative tumor size in the liver (< 50%/≥ 50%)	47/8
TACE refractory (without/with)	42/13
Extrahepatic spread (without/with)	49/6
HCC stage (III/IVa/IVb)^b^	30/22/3
BCLC stage (B/C)^c^	18/37
AFP (ng/mL)	1895.2 (2.6–529,500)
DCP (mAU/mL)	3854 (24–226,990)

Abbreviations: *AFP* alpha-fetoprotein, *DCP* des-gamma-carboxy prothrombin, *ECOG* Eastern Cooperative Oncology Group, *HBV* hepatitis B virus, *HCC* hepatocellular carcinoma, *HCV* hepatitis C virus, *Vp* portal invasion, *Vv* venous invasion

^a^Vp0 through Vp4 indicated no, third branch, second branch (segmental invasion), first branch (branch invasion) and main portal vein invasion, respectively, according to Liver Cancer Study Group of Japan criteria

^b^According to the Liver Cancer Group of Japan

^c^BCLC: Barcelona Clinic Liver Cancer,

### Efficacy

**Fig. 2:**
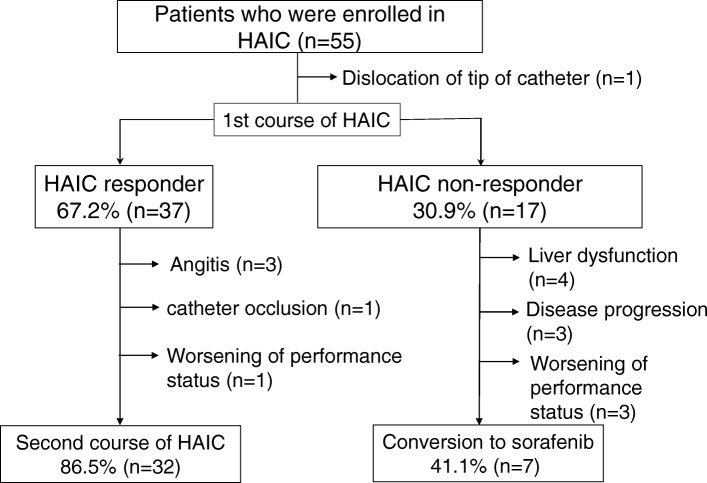
Patient flow chart. Abbreviations: *HAIC* hepatic arterial infusion chemotherapy

Figure 2 shows the flow of patients through the study. The number of responders was 37 patients (68.5%), and the number of non-responders was 17 patients (30.9%). Among the responders, 32 patients received a second course of HAIC. Five patients could not undergo the second course because of angitis, catheter occlusion, or worsening of performance status. Among the non-responders, 7 patients switched to sorafenib, whereas 10 patients were ineligible for sorafenib treatment due to liver dysfunction, disease progression, or worsening of performance status. The imaging response by the Response Evaluation Criteria In Solid Tumors to the first course of treatment was CR in one (1.8%) patient, PR in 13 (23.6%), SD in 30 (54.5%), and PD in 10 (18.1%) patients. SD patients were classified into two groups: 23 patients (41.8%) had SD with AFP ratio < 1 or DCP ratio < 1, whereas 7 (12.7%) had SD with AFP ratio > 1 and DCP ratio > 1.

### Survival

Among 55 patients, 27 patients died of HCC; no patients died of other diseases.

**Fig. 3:**
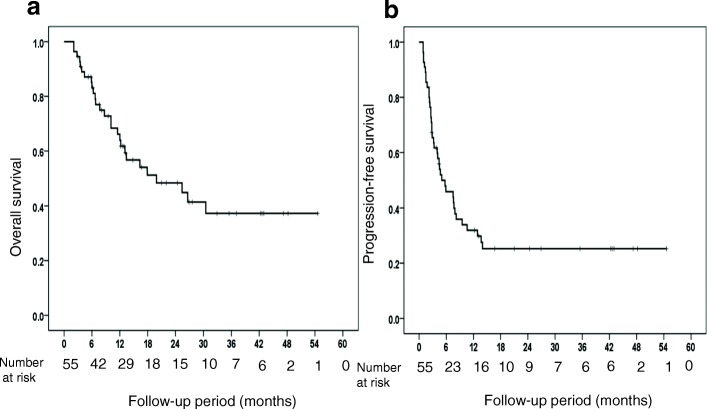
(**a**) Overall survival (**b**) Progression free survival

In the intent-to-treat population, the 1-year and 2-year survival rates were 64.0 and 48.3%, respectively (Fig. 3a). The median survival time was 19.9 months, and the PFS of the responders to HAIC was 5.0 months (Fig. 3b).

**Fig. 4:**
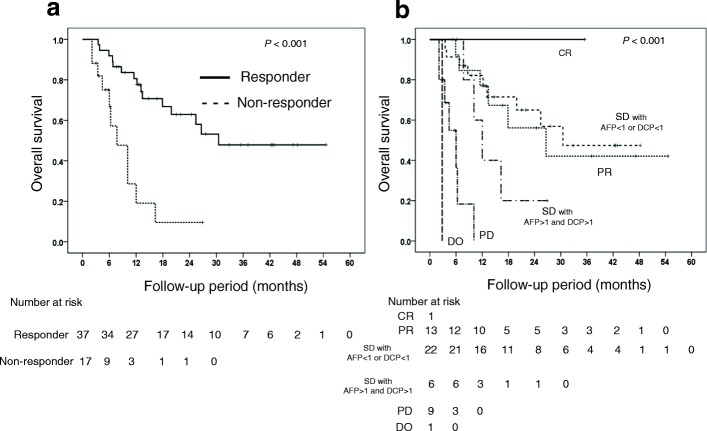
(**a**) Overall survival according to response (**b**) Overall survival according to responder or non-responder status

The MST of the responders to HAIC and of the non-responders to the first course of HAIC were 30.5 and 7.7 months, respectively. MST differed significantly between the responders and non-responders (*P* < 0.001). In the responders, the 1-year and 2-year survival rates were 78 and 62%, respectively. In the non-responders, the 1-year and 2-year survival rates were 28 and 12%, respectively (Fig. 4a).

MST differed significantly among the imaging response groups (*P* < 0.0001): 26.6, 30.5, 12.0, and 6.0 months in patients with PR, SD (AFP ratio < 1 or DCP ratio < 1), SD (AFP ratio > 1 and DCP ratio > 1), and PD, respectively (Fig. 4b).

### Safety profile

Adverse events (AE) during the first course of HAIC are shown in Table 2. The most common AEs were anemia, platelet count decrease, AST/ALT increase and leucocyte count decrease. However, the frequency of AE ≥ grade 3 was 21.8%.

**Table 2:** Adverse events associated with the first course of hepatic arterial infusion chemotherapy

No. (%)	Grade 1	Grade 2	Grade 3	Grade 4	Total
Clinical					
Nausea/Vomiting	9 (17)	2 (4)	0	0	11(20)
Anorexia	10 (19)	7 (13)	1(25)	0	18 (33)
Fever	5 (9)	1 (25)	0	0	6 (11)
Pain	11 (20)	0	0	0	11 (20)
Fatigue	8 (15)	1(25)	0	0	9 (17)
Diarrhea	0	1(25)	0	0	1(25)
Laboratory abnormalities					
Leucocyte count decrease	22 (41)	6 (11)	1(25)	0	29 (54)
Neutrophil count decrease	10 (19)	2 (4)	0	0	12 (22)
Anemia	26 (48)	7 (13)	2 (4)	0	35 (65)
Platelet count decrease	22 (41)	9 (17)	4 (7)	0	35 (65)
AST/ALT increase	24 (44)	4 (7)	4 (7)	0	32 (59)
Creatinine increase	15 (28)	0	0	0	15 (28)
Total			12 (21.8)	0 (0)	

Abbreviations: *ALT* alanine aminotransferase, *AST* aspartate aminotransferase

### Predictive parameters of efficacy and overall survival

The univariate analysis identified three parameters that were correlated either significantly or marginally with response: TACE refractory status (without TACE refractory; *P* = 0.007), and MVI (without MVI; *P* = 0.018). TACE refractory status and MVI were entered into the multiple logistic regression analysis to identify significant independent predictive factors. The multivariate analysis identified the without-TACE refractory stratus as the only significant and independent factor that influenced response (Table 3).

**Table 3:** Univariate and multivariate analyses of factors associated with response

Parameters	Univariate analysis	Multivariate analysis		
	*P* value	Odds ratio	95% CI	*P* value
Age (< 65/≥ 65 years)	0.462			
Gender (Male/Female)	0.917			
ECOG performance status (0/1)	0.667			
Platelet count (< 14.9 × 10^4^/> 14.9 × 10^4^ /μL)	0.487			
Child-Pugh score (A/B)	0.736			
Diameter of main tumor (< 80 mm/≥ 80 mm)	0.52			
Macroscopic vascular invasion (without/with)	0.018			
TACE refractory (without/with)	0.007	5.689	1.490–21.724	0.011
AFP (< 1895/≥ 1895 ng /mL)	0.149			
DCP (< 3854/≥ 3854 mAU/mL)	0.547			
Extrahepatic spread (without/with)	0.3			

Abbreviations: *AFP* alpha-fetoprotein, *DCP* des-gamma-carboxy prothrombin, *ECOG* Eastern Cooperative Oncology Group, *MVI* macroscopic vascular invasion, *TACE* transarterial chemoembolization

By means of univariate analysis, we then investigated the relationship between survival after the initiation of HAIC treatment and various clinicopathological variables (Table 4). Child-Pugh A, platelet count, DCP and EHS correlated significantly with OS. The above parameters were then entered into a multiple Cox proportional-hazard model analysis. This analysis identified EHS as a significant and independent determinant of survival.

**Table 4:** Univariate and multivariate analyses for determinants of overall survival

Parameters	Univariate analysis	Multivariate analysis		
	*P* value	Hazard ratio	95% CI	*P* value
Age (< 65/≥65 years)	0.304			
Gender (Male/Female)	0.325			
ECOG performance status (0/1)	0.187			
Platelet count (< 14.9 × 10^4^/> 14.9 × 10^4^ /μL)	0.07			
Child-Pugh score (A/B)	0.008			
Diameter of main tumor(< 80 mm/≥ 80 mm)	0.036			
Macroscopic vascular invasion (without/with)	0.646			
TACE refractory (without/with)	0.101			
AFP (< 1895/≥ 1895 ng /mL)	0.515			
DCP (< 3854/≥ 3854 mAU/mL)	0.055			
Extrahepatic spread (without/with)	0.004	3.905	1.420–10.736	0.008

Abbreviations: *AFP* alpha-fetoprotein, *DCP* des-gamma-carboxy prothrombin, *ECOG* Eastern Cooperative Oncology Group, *MVI* macroscopic vascular invasion, *TACE* transarterial chemoembolization

**Fig. 5:**
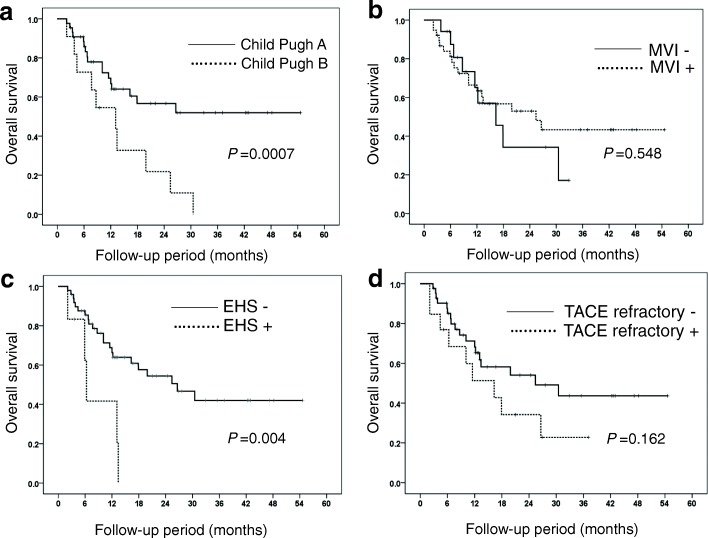
(**a**) Overall survival according to Child Pugh grade (**b**), macroscopic vessel invasion (MVI), (**c**) extrahepatic spread (EHS), and (**d**) transcatheter arterial chemoembolization (TACE) refractory

Subgroup analysis was performed according to Child-Pugh status, macroscopic vessel invasion, EHS and TACE refractory status. MST (25 months) of Child-Pugh A patients was significantly longer than that (13 months) of Child-Pugh B patients (*P* = 0.0007) (Fig. 5a). The MST of patients who had HCC with and without macroscopic vessel invasion were not significantly different: 25.4 months and 16.3 months, respectively (Fig. 5b). The MST of patients who had HCC without EHS was significantly longer than that of patients who had HCC with EHS (26.6 vs 6.3 months, respectively) (*P* < 0.001) (Fig. 5c). The MST of patients without and with TACE refractory status was not significantly different: 25.4 months and 16.3 months, respectively (Fig. 5d).

## Discussion

We investigated the efficacy of a protocol in which HAIC was selected as the first-line therapy for patients with advanced HCC and sorafenib was selected as the second-line therapy for patients refractory to HAIC. In our study, the 1-year and 2-year survival rates were 64.0 and 48.3%, and the MST was 19.9 months. OS was judged to be favorable with HAIC as first-line therapy for patients with advanced HCC. The rate of AEs (grade ≥ 3: 21.8%) was judged to be acceptable by the investigators.

Sorafenib is currently the standard first-line therapy for advanced HCC patients. However, the MST and response rate were almost 10 months and 10% with sorafenib therapy, respectively. In addition, HAIC is not used as a standard therapy for advanced HCC patients due to the lack of clinical trial data supporting its use.

The primary endpoint of the 1-year survival rate was 64.0%, and the MST was 30.5 months. When we compared our protocol to other treatment protocols for advanced HCC, the 1-year survival rates in the SHARP study and in the Asia-Pacific study of sorafenib monotherapy were 44 and 32%, respectively [13, 14]. HAIC therapy followed by sorafenib was superior to sorafenib monotherapy. In subgroup analysis of our study, the MST of patients who had HCC with and without macroscopic vessel invasion was not significantly different: 25.4 months and 16.3 months, respectively (Fig. 5b). The MST of patients who had HCC without EHS was significantly longer than that of patients who had HCC with EHS (26.6 vs 6.3 months, respectively) (*P* < 0.001) (Fig. 5c). Bruix et al. reported that the MST of patients who had HCC with and without macroscopic vessel invasion were 8.1 months and 14.1 months, respectively, in sub-analysis of the SHARP study [21]. Cheng et al. reported that the MST of patients who had HCC with macroscopic vessel invasion and/or EHS was 5.6 months, and the MST of patients who had HCC without macroscopic vessel invasion or EHS was 14.3 months, respectively, in sub-analysis of the Asia-Pacific trial. Therefore, HAIC therapy followed by sorafenib was superior to sorafenib monotherapy in patients with macroscopic vessel invasion. HAIC therapy followed by sorafenib was not inferior to sorafenib monotherapy in patients with EHS [21]. Furthermore, we compared our protocol to a previous HAIC study. Nouso et al. reported that the 1-year survival rate of HAIC was 52% in a nationwide study in Japan [22]. Although, there were few differences between our protocol and Nouso’s study, results of HAIC therapy followed by sorafenib in our study was superior to that of the previous HAIC study. The reason for our favorable results could be that we continued HAIC in HAIC responders, who are expected to have good prognoses, and switched to sorafenib therapy in HAIC non-responders, avoiding unnecessary AEs associated with HAIC.

In our study, 21.8% of patients had AE ≥ grade 3. Similar to our study, rates of AE ≥ grade 3 were 36 and 23.5% in the SHARP and Asia-Pacific studies, respectively [13, 14]. While occlusion of catheter and angitis as HAIC-specific AEs were observed in our study, the rates of HAIC-specific AEs in this study were similar to those of previous studies [11, 12].

Our multivariate analysis identified TACE non-refractory status as the only significant and independent factor that influenced response. In addition, a multiple Cox proportional-hazard model analysis identified lack of EHS as a significant and independent determinant for OS. Retrospective studies have shown similar results. In two studies, OS was significantly longer in those treated with sorafenib compared with HAIC in HCC patients refractory to TACE. A possible reason is that those studies involved shorter duration of HAIC and a need to withdraw the treatment due to stenosis of hepatic artery by catheter therapy, reduced sensitivity to the drug, deterioration of liver function, and appearance of collateral arteries [23, 24]. Another study reported that EHS was a poor prognosis factor in HAIC therapy [25]. Given these results, if our study protocol were to be conducted in patients with TACE non-refractory status and without EHS, favorable results are likely. This protocol should therefore be taken into consideration in the study design of a future clinical trial.

The study had several limitations: it was a single-arm study with a small sample size and a narrow period of observation. While we need to follow the prognosis over a longer time period, the results of this prospective study show the usefulness of this protocol as a first-line therapy for patients with advanced HCC. Larger comparative studies are necessary to confirm this conclusion.

## Conclusion

We found favorable outcomes in patients with advanced HCC treated with HAIC as first-line therapy. Given the results of this study, this protocol deserves consideration as an optional therapy for advanced HCC patients in the future.

## Abbreviations

AE: Adverse event
CTCAE: Common Terminology Criteria for Adverse Events
HAIC: Hepatic arterial infusion chemotherapy
HCC: Hepatocellular carcinoma
HICS: Hepatic Arterial Infusion Chemotherapy followed by Sorafenib
MST: Median survival time
OS: Overall survival
PFS: Progression-free survival
TACE: Transcatheter arterial chemoembolization
